# Silica removal at sewage treatment plants causes new silica deficiency

**DOI:** 10.1038/s41598-022-12272-8

**Published:** 2022-05-17

**Authors:** Atsushi Kubo, Koki Kawarasaki, Hiroshi Hara

**Affiliations:** 1 Department of Geosciences, Shizuoka University, 836 Ohya, Suruga-ku, Shizuoka, 422-8529 Japan; 2 Sewer Water Quality Section, Water Quality Management Division, Waterworks Department, Waterworks and Sewerage Bureau, Shizuoka City Hall, Shizuoka, Japan

**Keywords:** Element cycles, Marine chemistry, Biogeochemistry, Environmental sciences, Limnology, Ocean sciences

## Abstract

The dissolved silicate (DSi) concentration in coastal waters has decreased due to anthropogenic activities. Many studies have indicated that dam construction is a main reason for this reduction. However, recently, dam construction alone has not been sufficient to explain the DSi reduction in some coastal waters. In this study, we focused on silica removal at sewage treatment plants (STPs). DSi and particulate silica (PSi) concentrations were measured in STP influent and effluent waters from September 2020 to September 2021. Dissolution experiments on PSi were also conducted to estimate the fraction of soluble PSi in the STP influent. DSi and PSi were removed by 29.5% and 96.9%, respectively, at the STP. In addition, the soluble PSi in the STP influent accounted for 20.3% of the PSi removed. Therefore, in addition to the DSi removal in STPs, removal of soluble PSi can also cause potential DSi depletion in downstream and coastal waters. In addition to the effect of dams, the silica supply delivered to coastal waters may be further reduced in the future due to the progress of sewage treatment development in coastal areas.

## Introduction

Dissolved silicate (DSi) in rivers and coastal waters is essential for some aquatic plants and organisms. For example, diatoms, which play a significant role as primary producers in marine ecosystems, require DSi to produce silicate shells^1^. Primary production derived from diatoms accounts for approximately 45% of the total primary production in the ocean^2,3^. When the DSi concentration in the water decreases, the growth of diatoms is suppressed. Consequently, the development of non-diatom taxa, such as dinoflagellates, is promoted, resulting in the replacement of primary producers^4^. Dinoflagellates are responsible for harmful algal blooms and shellfish poisoning. Therefore, it is essential to understand the current status of DSi concentrations in the ocean to consider the ecosystems of coastal waters.

The most significant anthropogenic sink for DSi is artificial dams^5^. The damming of the upper Danube River increased the water residence time behind the dam and decreased the DSi load delivered downstream due to diatom blooms and particulate silica (PSi) sedimentation (silica deficiency hypothesis). In contrast, dissolved inorganic nitrogen and phosphate are also used for growth, but these are then quickly decomposed; therefore, most of these elements are supplied to regions downstream. Therefore, in the Black Sea, where the Danube River debouches, DSi has decreased relative to nitrogen, and phosphorus and non-silica algae have increased^6^. Since the report of Humborg et al.^5^, silica deficiency attributable to the construction of dams has been reported in coastal waters worldwide (e.g.,^7,9^). However, a decrease in the DSi concentration in Tokyo Bay has been reported in recent years, and dam construction cannot explain this decrease^10^.

The number of sewage treatment plants (STPs) has increased with the rapid urbanization of coastal areas^11^. However, although many studies have considered the removal of dissolved inorganic nitrogen and phosphate in STPs (e.g.,^12,13^), none have focused on DSi. Maguire and Fulweiler^14^ estimated DSi removal for the first time based on the change in the DSi concentrations between STP influent and effluent throughout the year at the Deer Island STP in the United States. They concluded that there was no significant DSi removal or supply at the STP. However, this is the only study in which silica removal by an STP has been estimated. In addition, Maguire and Fulweiler^14^ did not evaluate the reduction in PSi. Although phytoplankton cannot utilize PSi directly, it can be a potential source of DSi if PSi dissolves rapidly.

In this study, we collected influent and effluent from the Nakajima STP in the city of Shizuoka throughout the year to evaluate the influence of STPs on the silica cycle in the river and coastal waters quantitatively. We estimated the amounts of DSi and PSi removed by the STP. The dissolution characteristics of PSi were also evaluated by conducting dissolution experiments using the STP influent.

## Methods

The observation was conducted at the Nakajima STP in Shizuoka (34°56′06.7" N 138°23′50.7" E), which includes sewerage of separate systems, and was conducted up to the secondary treatment stage. Samples were collected from the STP influent and effluent to estimate the amounts of DSi and PSi removed. Forty-six observations were carried out through the one year from September 2020 to September 2021. The collected water was filtered through a nucleopore filter (PC MB, pore size 0.6 µm, Whatman, UK), the filtrate was used for DSi analysis, and the filter was used as the sample for PSi analysis. Samples for the PSi dissolution experiments were collected from STP influent. For the dissolution experiments, the STP influent was filtered through a 2 mm mesh stainless steel sieve. Then, 1.0 L of filtered STP influent, 1.0 mL of HgCl_2_, and a stirrer bar were placed in a polyethylene beaker and incubated with Parafilm to prevent evaporation to the greatest extent possible. The dissolution experiments were conducted in the incubator at 22℃ for one week with a sample stirring in a stirrer to agitate the suspended material to prevent it from sinking. The experiment was carried out in quadruplicate. The dissolution amount was estimated from the change in the DSi and PSi concentrations from before to after incubation. The rate of change in DSi and PSi concentration from before to after the incubation experiment was shown in ΔDSi and ΔPSi, respectively. All DSi and PSi analyses were conducted according to Kubo and Yamahira using a spectrophotometer with a syringe shipper unit (UVmini1240, Shimadzu, Japan)^15^.

The annual loadings of DSi and PSi at the STP were estimated using Beal's unbiased ratio estimator, which is ideally suited to situations in which flow rate information is abundant but there is relatively little information on concentration^14,16^.

## Results and discussion

**Figure 1:**
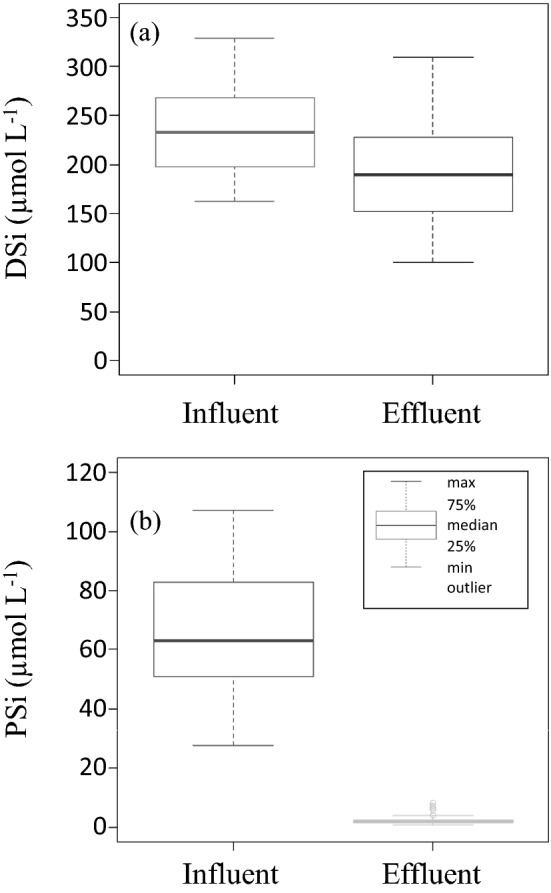
Box plots of annual average (**a**) DSi and (**b**) PSi concentrations (µmol L^-1^) in STP influent and effluent. For each box plot, the median is represented by the bolded line while the 25th and 75th percentile are represented by the bottom and top of the box, respectively. The whiskers represent the minimum and maximum values (1.5 × interquartile range), while outliers are plotted as circles.

**Figure 2:**
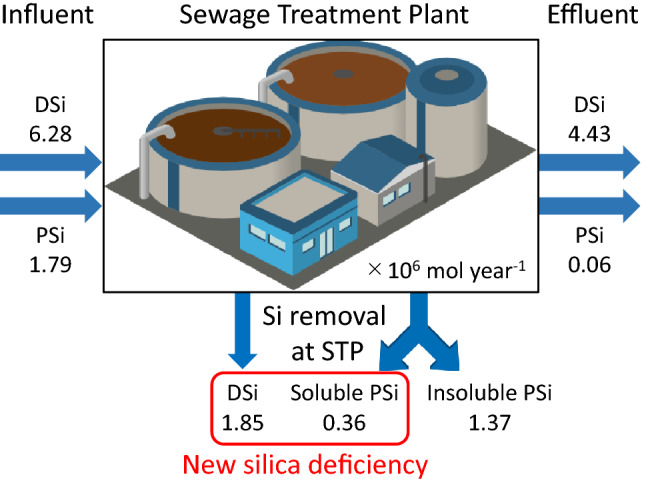
Si cycling model in an STP. Each line shows the Si amount of STP influent, effluent, and removal, respectively. The reduction of DSi and soluble PSi by the STP creates a new silica deficiency hypothesis (the illustration was created by the image library, free material provided by the Integration and Application Network, University of Maryland Center for Environmental Science [ian.umces.edu/imagelibrary/]).

The annual mean DSi concentrations of the influent and effluent at the STP were 235.4 ± 42.8 µmol L^−1^ (annual mean concentration ± standard deviation) and 193.9 ± 47.6 µmol L^−1^, respectively (Fig. 1). The DSi concentration of the effluent was significantly lower than the concentration of the influent in 40 of the 46 observations (*t*-test, *p* < 0.0001). The annual mean DSi influent and effluent loads at the observation date were 16,959 ± 3596 mol day^−1^ and 12,093 ± 3542 mol day^−1^, respectively, and the effluent had a significantly lower load than the influent (*t*-test, p < 0.0001). The annual DSi loadings were 6.28 × 10^6^ mol year^−1^ for influent and 4.43 × 10^6^ mol year^−1^ for effluent (Fig. 2). In contrast, the annual mean PSi concentrations of the influent and effluent at the STP were 67.7 ± 23.9 µmol L^−1^ and 2.5 ± 1.6 µmol L^−1^, respectively (Fig. 1). Unlike the DSi concentration, the PSi concentration of the effluent was significantly lower than the concentration of the influent in all observations (*t*-test, *p* < 0.0001). The annual mean PSi influent and effluent loads at the observation date were 4863 ± 1772 mol day^−1^ and 153 ± 104 mol day^−1^, respectively, and the effluent had a significantly lower value than the influent (*t*-test, *p* < 0.0001). Therefore, the PSi, as in the case of DSi, decreased considerably at the STP. The annual PSi loadings were 1.79 × 10^6^ mol year^−1^ for influent and 5.61 × 10^4^ mol year^−1^ for effluent (Fig. 2). The annual removal rates of DSi and PSi were 29.5 and 96.9%, respectively. PSi was, as a matter of course, removed by precipitation during primary treatment at the STP. However, previous studies have shown that DSi concentration and loading do not decrease significantly during primary and secondary treatment in STPs^14^.

Two possible reasons for the decrease in DSi concentration at the STP of this study are physicochemical and biological removal. The coprecipitation of DSi with metal ions, such as magnesium, aluminum, and iron ions, is one factor. Magnesium hydroxide has generally been reported to precipitate particles most efficiently under alkaline conditions^17^. In addition, metal ions, such as magnesium, aluminum, and iron, at pH 6 and 9, accelerate the polymerization of DSi^18^. Therefore, the coprecipitation of DSi with metal ions, which are high in STP influent, may have reduced the DSi concentration of the effluent. Magnesium hydroxide is generally used to neutralize sulfur oxides and neutralization of wastewater at factories. However, excessive magnesium hydroxide addition may be causing inflow into the STP.

The second factor, biological removal, consists of the uptake of DSi by bacteria of genus *Bacillus*. Spores of species of this genus have a layered structure to protect the nucleic acids and proteins, with a layer of nano-sized particulate matter outside the coat layers^19^. DSi is incorporated into these layers^19^. *Bacillus* species are ubiquitous in a wide variety of habitats, including soil^20^, freshwater^21^, and marine sediments^22^. In addition to these natural environments, *Bacillus* spp. are present in the activated sludge used for secondary sewage treatment at STPs^23,24^. *Bacillus* takes up 29 µmol L^−1^ h^−1^ of DSi during growth^19^. According to Murakami et al.^25^ *Bacillus* spp. account for 92–98% of the total bacteria (5 × 10^7^ to 5 × 10^10^ cells mL^−1^) in the activated sludge of secondary treatment. In particular, *Bacillus thuringiensis* is indispensable in STPs because of its ability to degrade starch and oil^26^. These results suggest that *Bacillus* bacteria living in the secondary treatment environment of STPs take up DSi and precipitate it in the sewage influent as sludge, thereby reducing the DSi load in the effluent.

This study does not know whether physicochemical or biological removal substantially influences DSi reduction in STP. The physicochemical removal occurs during the primary treatment process, while the biological removal occurs during the secondary treatment process. Therefore, it is possible to identify the primary factors by evaluating the changes in DSi concentrations during each treatment process. Furthermore, it is still unclear what kind of changes occur at STPs that perform the advanced treatment process. In addition, the reason why DSi reduction in some STP (this study) and not the other^14^ was unclear (e.g., a difference in pH, alkalinity, and activated sludge quality). We need to pay more attention to the DSi changes in STPs and evaluate them in more detail.

In the PSi dissolution experiment using STP influent (Table 1), the DSi concentration increased by an average of 8.1 ± 6.8% and the PSi concentration decreased significantly by an average of 20.3 ± 7.5%. The total Si concentration (DSi + PSi) was 304.0 ± 35.2 µmol L^−1^ before the incubation and 297.2 ± 42.6 µmol L^−1^ at the end of incubation, indicating no significant difference. The dissolution experiments suggest that some of the PSi entering the STP is soluble, approximately 20.3%. Therefore, the soluble PSi loading in the STP influent would be approximately 0.36 × 10^6^, and most of this soluble PSi would be expected to be removed by the STP. This amount is comparable to the 20.0% of DSi removal amount of 1.85 × 10^6^ mol year^−1^ by the STP (Fig. 2). Therefore, the removal of DSi and soluble PSi attributable to constructing the STP might have reduced the DSi load in the watershed by 2.21 × 10^6^ mol year^−1^.

**Table 1:** Results of the PSi dissolution experiment. The results at time 0 and the time of 7 days indicate the concentrations of STP influent before and after the incubation experiment, respectively.

	DSi (µmol L^−1^)		PSi (µmol L^−1^)		TSi (µmol L^−1^)		**Δ**DSi%	**Δ**PSi%
	Time 0	7 days	Time 0	7 days	Time 0	7 days		
Incubation 1 (6/2/2021)	214.5	257.1 ± 10.0	149.9	109.4 ± 16.4	364.4	366.5 ± 15.7	+ 19.9	− 27.0
Incubation 2 (6/8/2021)	219.2	229.3 ± 2.9	72.1	66.7 ± 3.8	291.2	296 ± 1.7	+ 4.6	− 7.5
Incubation 3 (6/8/2021)	212.5	220.5 ± 1.9	66.3	51.0 ± 5.5	278.9	271.5 ± 3.7	+ 3.8	− 23.2
Incubation 4 (6/8/2021)	222.2	231.4 ± 3.1	59.2	45.4 ± 1.2	281.5	276.8 ± 2.5	+ 4.1	− 23.4
Average	217.1 ± 3.8	234.6 ± 13.6	86.9 ± 36.7	62.6 ± 31.1	304.0 ± 35.2	297.2 ± 42.6	8.1 ± 6.8	− 20.3 ± 7.5

ΔDSi and ΔPSi show the rate of change in DSi and PSi concentration from before to after the incubation experiment.

In this study, the DSi and PSi loadings decreased due to the progress of sewage maintenance in the watershed. Thus, the future construction of STPs in coastal watersheds causes a "new silica deficiency hypothesis" for coastal waters around the world (Fig. 2). However, at present, time-series observations of DSi in many coastal waters are less available than those of nitrogen and phosphorus. Therefore, data on DSi, as an essential parameter for environmental change, should be accumulated continuously from the present time before any changes occur.

## Conclusions

In this study, both DSi and PSi were significantly removed by the STP (26.5% and 96.9%, respectively). The removal of DSi can be attributed to coprecipitation with metal ions contained in the sewage effluent and/or to uptake by bacteria universally present in the activated sludge. In addition, the soluble PSi in the STP influent accounted for about 20.3% of the PSi removed. The removal of DSi and soluble PSi attributable to the progress of sewage maintenance worldwide could cause new silica deficiency problem in coastal waters.

## Data Availability

The datasets of DSi and PSi during the current study are available from the corresponding author on reasonable request.
